# Prevalence and factors associated with pulmonary arterial hypertension on maintenance hemodialysis patients in Kinshasa, Democratic Republic of Congo: a cross-sectional study

**DOI:** 10.1186/s12882-020-02131-x

**Published:** 2020-11-04

**Authors:** Yannick Mompango Engole, François Bompeka Lepira, Yannick Mayamba Nlandu, Yves Simbi Lubenga, Augustin Luzayadio Longo, Aliocha Nkodila, Jean-Robert Rissassy Makulo, Vieux Momeme Mokoli, Justine Busanga Bukabau, Marie-France Ingole Mboliasa, Evariste Mukendi Kadima, Cedric Kabemba Ilunga, Tresor Swambulu Mvunzi, Nazaire Mangani Nseka, Ernest Kiswaya Sumaili

**Affiliations:** 1 Nephrology Unit, University Hospital of Kinshasa, Kinshasa, Democratic Republic of the Congo; 2 Cardiology Unit, University Hospital of Kinshasa, BP: 123, Kinshasa, XI Democratic Republic of the Congo

**Keywords:** Pulmonary hypertension, Hemodialysis, Systolic pulmonary arterial pressure, Cardiovascular disease

## Abstract

**Background:**

Although cardiovascular diseases in particular Pulmonary Arterial Hypertension (PAH) is associated with, high morbid-mortality in chronic hemodialysis, but its magnitude remains paradoxically unknown in sub-Saharan Africa. The aim of this study was to evaluate the prevalence of PAH and associated factors in chronic hemodialysis in Sub-Saharan African population.

**Method:**

In a cross-sectional study, patients treated with HD for at least 6 months in 4 hemodialysis centers were examined. PAH was defined as estimated systolic pulmonary arterial pressure (sPAP) ≥ 35 mmHg using transthoracic Doppler echocardiography performed 24 h after the HD session.

**Results:**

Eighty-five HD patients were included; their average age was 52.6 ± 15.9 years. Fifty-seven patients (67.1%) were male. Mean duration of HD was 13.3 ± 11 months. With reference to vascular access, 12 (14.1%), 29 (34.1%) and 44 (51.8%) patients had AVF, tunneled cuff and temporary catheter, respectively. The underlying cause of ESRD was diabetes in 30 patients (35.3%). The prevalence of PAH was 29.4%. Patients with PAH had more hyponatremia (11 (44%) vs 10 (16.7%), *p* = 0.010). In multivariate analysis, unsecured healthcare funding (aOR 4, 95% CI [1.18–6.018]), arrhythmia (aOR 3, 95% CI [1.29–7.34]), vascular access change (aOR 4, 95% CI [1.18–7.51]) and diastolic dysfunction (aOR 5, 95% CI [1.35–9.57] were independently associated with PAH.

**Conclusion:**

One third of hemodialysis patients exhibit PAH, which is independently associated with low socioeconomic status (unsecured funding, vascular access change) and cardiovascular complications (arrhythmia, diastolic dysfunction).

## Background

Excessive cardiovascular mortality in patients with end-stage renal disease (ESRD) has been described in epidemiological and clinical studies. It accounts for about 50% of deaths in dialysis [1] with a relative excess mortality risk of 2.4 [2]. Pulmonary arterial hypertension (PAH), defined as a rise in pulmonary arterial pressure (PAP) resulting from heart, lung or systemic disorders, is a common finding in patients on maintenance hemodialysis [3, 4] and an independent predictor of all-causes and cardiovascular mortality in maintenance hemodialysis patients [5–8]. The prevalence of PAH varies from 23 to 33%, as detected by Doppler echocardiography in patients on chronic hemodialysis (HD). Several studies have reported PAH-related mortality: In this regard, the Jackson Heart Study has indicated that PAH was associated with higher risk for heart failure hospitalization and mortality multiplying the Hazard ratio (HR) by 2.37 and 1.84, respectively [9]. In addition, Tang et al. [10] showed that PAH was associated with increased risk for cardiovascular events in patients with CKD (RR 1.67) and ESRD receiving dialysis (RR 2.33). In these patients, PAH may lead to enlarged left atrium, altered arteriovenous fistula (AVF) flow, elevated thromboxane β2 and left ventricular diastolic dysfunction [11]. Although the pathogenic mechanism of PAH remains unclear, several factors may cause PAH such as endothelial dysfunction, vascular calcification, diastolic dysfunction, AVF flow, volume overload, left ventricular hypertrophy, dialysis membrane exposures, obstructive sleep apnea syndrome, and pulmonary embolism [12].

To date, there has been no study assess the burden of PAH on maintenance HD from Sub-Saharan Africa (SSA). Yet, early intervention to reduce pressure in the pulmonary artery can prevent the worsening of heart failure and death [13–16]. Therefore, the present study aimed is to evaluate the prevalence and associated factors of PAH in chronic hemodialysis in Sub-Saharan African population.

## Methods

### Study design and setting

A cross-sectional study was conducted, between Mars 2016 and June 2019, in 4 hemodialysis centers (Kinshasa University Hospital, Ngaliema Medical center, Afia Medical Care, Medical Center of Kinshasa).

### Population study and eligibility criteria

**Fig. 1:**
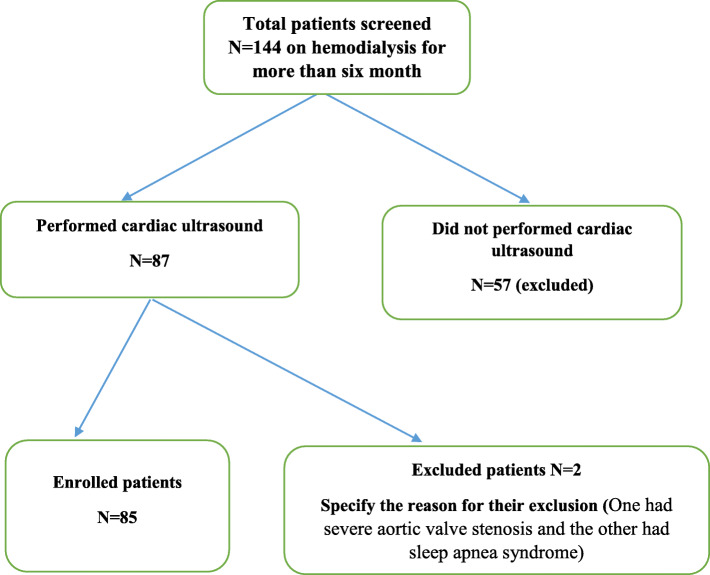
Flow chart of the study population

All consecutive ESRD patients aged > 18 years old on maintenance HD for more than 6 months were eligible to participate in this study (Fig. 1). To control potential sources of bias with PAH, patients on chronic hemodialysis with the following characteristics were excluded: chronic pulmonary diseases such as chronic obstructive pulmonary disease, pulmonary fibrosis, pregnant patients, chest wall, previous pulmonary embolism, collagen vascular disease, moderate or severe mitral or aortic valve disease, severe heart failure and having obstructive sleep apnea syndrome.

### Hemodialysis parameters

Patients had two or three sessions per a week for 24 h with anticoagulant (low molecular weight heparin or unfractionated heparin) at each session. Most patients were treated with synthetic dialyzer membranes (high flux) and bicarbonate-based on dialysis solution upon a bicarbonate concentration of 32 mEq/L. Blood flow and dialysate flow rate varied between 200 and 400 mL/min and 500–800 mL/min, respectively using 4008 and 5008 s generators of Fresenius.

### Echocardiographic measurements

Transthoracic Doppler echocardiograms were performed by a single cardiologist using an ultrasound system with a 3.5 Hz cardiac lead (Vivid 7, GE, Massachusetts, USA) as a non-invasive method, in post dialysis, 24 h after when patients were on optimal dry weight, PHT was defined as Systolic PAP equal to or greater than 35 mmHg. Systolic right ventricular (or pulmonary artery) pressure was calculated using the modified Bernoulli equation: PAP = tricuspid systolic jet (TR) + 10–15 mmHg (estimated right atrial pressure: 15 mmHg in dilated right atrium and 10 mmHg in normal or slightly enlarged right atrium) [17].

### Biological parameters

Laboratory investigations encompassed: blood urea nitrogen (BUN), serum creatinine, natremia, kaliemia, uric acid, hemoglobin, hematocrit, iron, ferritin, ProBNP, Troponin, calcium, phosphorus, and parathyroid hormone level. The results of the predialysis blood samples at the time of echocardiographic study and the mean of the preceding 3 months values were evaluated.

Others parameters of interest included the following: age, sex, comorbidities, medications, etiology of kidney disease, age at time of ESRD, source of funding, duration of hemodialysis therapy, and blood access location.

### Operational definitions

- ✓ Diastolic dysfunction: was defined as E/A < 1 or > 2 and between1–2, additionally with E/E’>15 [18].
- ✓ Systolic dysfunction: LVEF < 55%.
- ✓ Hypervolemia: water retention with tissue infiltration or clinical signs of hyperhydration
- ✓ Hyponatremia is an electrolyte disorder defined by a sodium concentration in the blood plasma of less than 136 mmol /l [19].
- ✓ Hypocalcemia: electrolyte disorder characterized by an adjusted total serum calcium level of < 8.4 mg/dl
- ✓ Arrhythmia: any rhythm disturbance objectified by the cardiologist during the echocardiography
- ✓ ESRD was defined as irreversible and advanced loss of kidney function due to any etiology requiring long term RRT with HD.
- ✓ Funding was considered secured when health insurance, social security, company or government provided financial support in HD, whereas unsecured healthcare funding as patient without secure financing.

### Statistical methods

All statistical analyses were performed with using SPSS (Statistic Package for Social Sciences) software for Windows version 24.

Data are presented as crude counts (n) and relative (%) frequencies for categorical variables, as an average (± standard deviation) for quantitative variables normally distributed, whereas non-normally distributed are presented as a median (Interquartile range: EIQ). Proportions were compared by the Pearson square Chi-square test or Fischer’s exact test as appropriate, whereas means or medians by the Student’s t test or the Man Whitney U test, respectively. Univariate logistic regression analysis was used to identify factors associated with PAH. In order to control the effect of potential confounding, we further performed multivariable logistic regression analysis (using ascending step-by-step approach) to search factors independently associated with PAH. The adjusted ORs and their 95% CI were calculated finally to determine the degree of association between the PAH and the independent variables. To search for risk factors, two multivariate mathematical models were used to limit the cumulative effect of independent variables. The significance level retained was then *p* < 0.05.

### Ethical issues

The research followed the tenets of the Declaration of Helsinki and informed consent was obtained. The Clinical Research Ethics Committee of Kinshasa School of Public Health (number ESP/CE/013/2016) approved the study protocol.

## Results

Eighty-five patients were enrolled in the study; 57 patients (67.1%) were male. Mean duration of HD was 13.3 ± 11 months. With reference to vascular access, 12 (14.1%), 29 (34.1%) and 44 (51.8%) patients had AVF, tunneled cuff and temporary catheter, respectively. The underlying cause of ESRD was diabetes in 30 patients (35.3%). Twenty-five (29.4%) patients (mean age 54.6 ± 14.3 years) had PAH with a mean value for SPAP of 28.0 ± 10 mmHg.

PAH was present in 25 (29.4%) patients. Patients with PAH had more hyponatremia (11 (44%) vs 10 (16.7%), *p* = 0.010), arrhythmia [4 (16%) vs 1 (1.7%), *p* = 0.025] and had lower β blockers [8 (32%) vs 9 (15%), *p* = 0.038] and Aspirin junior [15 (60%) vs 22 (36.7%), *p* = 0.041] than to those without PAH. They also did not have a secure funding [8 (32%) vs 36 (60%), *p* = 0.017] and experimented a vascular access change [18 (72%) vs 29 (48.3%), p = 0.038] (Table 1). Compared with patients without PAH, those with PAH had, in average, a significantly higher interdialytic weight gain [(2.8 ± 1.2) vs (3.4 ± 1.5) (*p* = 0.014)], pulse pressure [(67.3 ± 16.7) vs (82.2 ± 16.8) (*p* < 0.001)] (Table 2). These findings were similar to inferior vena cava diameter (*p* = 0.004), filling pressures E / E ‘(*p* = 0.001) and proportion of diastolic dysfunction (*p* = 0.007) (Table 3).

**Table 1:** Characteristics of HD patients according to presence of PAH

Variables	PAH- *n* = 60	PAH+ *n* = 25	***P-value***
Male, n (%)	39 (65.0)	18 (72.0)	0.359
Age ≥ 60 years, n (%)	22 (36.7)	13 (52.0)	0.143
Unemployed, n (%)	10 (16.7)	2 (8.0)	0.600
Secure funding	36 (60.0)	8 (32.0)	**0.017**
HT, n(%)	54 (90.0)	24 (96.0)	0.332
DM, n(%)	24 (40.0)	8 (32.0)	0.330
Tabacco, n(%)	6 (10.0)	4 (16.0)	0.329
Alcohol, n(%)	14 (23.3)	9 (36.0)	0.176
Hypervolemia, n(%)	31 (51.7)	11 (44.0)	0.343
Hyponatremia, n(%)	10 (16.7)	11 (44.0)	**0.010**
Hypocalcemia, n(%)	40 (66.7)	19 (76.0)	0.280
Vascular access, n(%)			0.248
Temporary catheter	34 (56.7)	10 (40.0)	
Permanent catheter	20 (33.3)	9 (36.0)	
Fistula	6 (10.0)	6 (24.0)	
HD session/week, n(%)			0.730
1	7 (11.7)	3 (12.0)	
2	26 (43.3)	13 (52.0)	
3	27 (45.0)	9 (36.0)	
Arrhythmia, n (%)	1 (1.7)	4 (16.0)	**0.025**
Vascular access change, n (%)	29 (48.3)	18 (72.0)	**0.038**
Supplement Ca/VitD, n (%)	31 (51.7)	14 (56.0)	0.451
Phosphorus chelator, n (%)	6 (10.0)	5 (20.0)	0.183
Diuretic, n (%)	30 (50.0)	16 (64.0)	0.173
EPO, n (%)	43 (71.7)	19 (76.0)	0.451
ACEI, n (%)	22 (36.7)	14 (56.0)	0.081
ARAII, n (%)	12 (20.0)	4 (16.0)	0.461
β-blockers, n (%)	9 (15.0)	8 (32.0)	**0.038**
Iron supplement, n (%)	45 (75.0)	22 (88.0)	0.147
Junior aspirin, n (%)	22 (36.7)	15 (60.0)	**0.041**
RUV, n (%)			0.343
< 500 ml	29 (48.3)	14 (56.0)	
≥ 500 ml	31 (51.7)	11 (44.0)	

*Abbreviations: HT* Hypertension, *DM* Diabetes mellitus, *HD* Hemodialysis, *Ca/VitD* Calcium and Vitamin D, *EPO* Erythropoeitin, *ACE* Angiotensin conversion enzyme inhibitor, *ARA* Angiotensin II receptor antagonist, *RUV* Residual urination volume

**Table 2:** Clinical, biological parameters according to presence of PAH

Variables	PAH-	PAH+	***P-value***
Age (years)	52.0 ± 16.5	54.6 ± 14.3	0.482
KT/V weekly	1.1 ± 0.2	1.1 ± 0.1	0.960
Dry weight, Kg	70.5 ± 16.3	66.9 ± 14.9	0.354
IWG max kg	2.8 ± 1.2	3.4 ± 1.5	**0.014**
Pre-dialysis SBP mmHg	153.0 ± 18.1	164.0 ± 18.2	**0.012**
Pre-dialysis DBP mmHg	85.6 ± 15.5	81.9 ± 14.8	0.311
PP mmHg	67.3 ± 16.7	82.2 ± 16.8	**< 0.001**
Duration in HD, month	14.4 ± 12.2	18.5 ± 10.2	0.264
BMI, kg/m^2^	25.5 ± 5.4	24.6 ± 4.6	0.503
Residual diuresis, mL	625.0 (241.0–1010.0)	774,0 (370.0–1680.0)	0.711
Creatinin, mg/dL	9.7 ± 3.2	9.9 ± 4.4	0.804
BUN, mg/dL	139.2 ± 51.9	147.3 ± 56.6	0.525
Uric Acid, mg/dL	7.3 ± 1.9	7.6 ± 2.0	0.560
Na^+^, mmol/L	134.7 ± 2.5	132.5 ± 3.4	**0.048**
K^+^, méq/L	4.6 ± 0.6	4.6 ± 0.6	0.949
PxCa,^2^ mg^2^/dL^2^	42.8 ± 12.9	44.5 ± 16.0	0.607
Albumin, g/L	36.3 ± 5.9	36.1 ± 5.4	0.876
Hb g/dL	9.0 ± 1.6	8.8 ± 1.0	0.509
Hct, %	27.2 ± 5.3	26.7 ± 3.6	0.672
Total Cholesterol, mg/dL	172.2 ± 38.7	183.7 ± 29.7	0.188
HDL-c, mg/dL	45.5 ± 17.1	50.1 ± 21.5	0.298
LDL-c, mg/dL	103.4 ± 33.3	118.4 ± 32.3	0.060
Triglyceride, mg/dL	114.2 ± 40.6	118.6 ± 37.2	0.642
Vit D, ng/mL	27,3 (0,0-35,0)	26.9 (10.4–38.5)	0.132
PTH, pg/mL	379,3 (197,6-509,0)	282.9 (16.1–1308.6)	0.886
Ferritin, ng/mL	339.5 (45.5–20,000)	458.4 (212.4–2000.0)	0.817
S. Iron, μmol/L	12.8 (10.2–14.5)	13.1 (9.5–24.9)	0.705
Troponin, ng/L	15.4 (1.5–48.0)	16.4 (7.6–34.0)	0.670
ProBNP, pg/mL	5907.0 (1509–22,222)	12,846.0 (4548–25,000)	0.425
CRP mg/L	33.3 (0.6–76.0)	18.5 (1.9–112.0)	0.186

Data are expressed as mean ± standard deviations (ET), crude (n) and relative frequency (in percent)

*Abbreviations: SBP* systolic blood pressure, *DBP* diastolic blood pressure, *PP* pulse pressure, *BMI* body mass index, *IWG* interdialytic weight gain, *eGFR* estimated glomerular filtration rate, *MDRD* modification of diet in renal disease, *BUN* blood urea nitrogen, *PxCa* phosphate calcium product, *PTH* parathormon, *HDL-c* high-density lipoprotein-cholesterol, *LDL-c* low-density lipoprotein-cholesterol, *ProBNP* Pro brain natriuretic peptid

**Table 3:** Echocardiography parameters according to presence PAH

Variables	PAH-	PAH +	***P value***
IVS	13.1 ± 2.4	13.6 ± 2.1	0.340
LVPW	11.8 ± 1.9	12,9 ± 2.8	**0.039**
LVEF	64.5 ± 8.6	64.3 ± 10.3	0.942
LVEDD	49.9 ± 6.1	50.4 ± 5.8	0.737
LVMi	369.4 ± 139.6	367.0 ± 170.9	0.948
ICV	13.6 ± 4.9	17.2 ± 5.6	**0.004**
E/A	1.0 ± 0.6	1.3 ± 0.9	0.118
E/E’	9.5 ± 4.1	14.0 ± 7.2	**0.001**
SPAP	23.1 ± 6.1	42.2 ± 6.9	**< 0.001**
DT	217.2 ± 45.9	243.4 ± 104.9	0.276
Left Atrium volume	20.6 ± 5.3	22.9 ± 6.4	0.080
Right Atrium volume	14.8 ± 3.0	18.2 ± 4.5	**< 0.001**
RVEDD	20.1 ± 6.3	21.2 ± 7.8	0.505
Calcifications	19 (31.7)	9 (36.0)	0.442
Systolic dysfunction	17 (28.3)	10 (40.0)	0.211
Diastolic dysfunction	8 (14.8)	6 (35.3)	**0.007**
Pericardial effusion	9 (15.0)	3 (12.0)	0.506

*IVS* interventricular septum, *LVPWd* left ventricular posterior wall in diastolic, *LVEF* left ventricular ejection fraction, *LVEDD* left ventricular end diastolic diameter, *LVMI* left ventricular mass index, *ICV* inferior cave veinous, *DT* deceleration time; *SPAP* systolic pressure arterial pulmonary

In logistic multivariate analysis (Table 4), unsecured healthcare funding (aOR 4, 95% CI [1.18–6.018]), arrhythmia (aOR 3, 95% CI [1.29–7.34]), vascular access change (aOR 4, 95% CI [1.18–7.51]) and diastolic dysfunction (aOR 5, 95% CI [1.35–9.57] were independently associated with PAH.

**Table 4:** Factors associated with PAH in univariate and multivariate logistic regression analysis

Variables	Univariate analysis		Multivariate analysis	
	*p*-value	unadjustedOR (IC95%)	*p*-value	aOR (IC95%)
**Model 1**				
Healthcare funding (no secured vs yes)	**0.021**	3.19 (1.19–8.55)	**0.026**	3.91 (1.18–6.010)
Hyponatremia (no vs yes)	**0.010**	3.93 (1.39–11.13)	0.085	2.81 (0.87–5.13)
Arrhythmia (no vs yes)	**0.035**	4.24 (1.19–6.33)	**0.030**	3.15 (1.29–7.34)
**Model 2**				
β-blockers (no vs yes)	**0.018**	2.67 (1.89–8.01)	0.251	1.98 (0.62–6.39)
Junior aspirin (no vs yes)	**0.015**	2.59 (1.99–6.75)	0.094	2.36 (0.86–6.44)
Vascular access change (no vs yes)	**0.041**	2.75 (1.02–7.54)	**0.034**	4.02 (1.18–7.51)
Diastolic dysfunction (no vs yes)	**0.007**	3.14 (2.90–10.91)	**0.011**	4.98 (1.35–9.57)

**Fig. 2:**
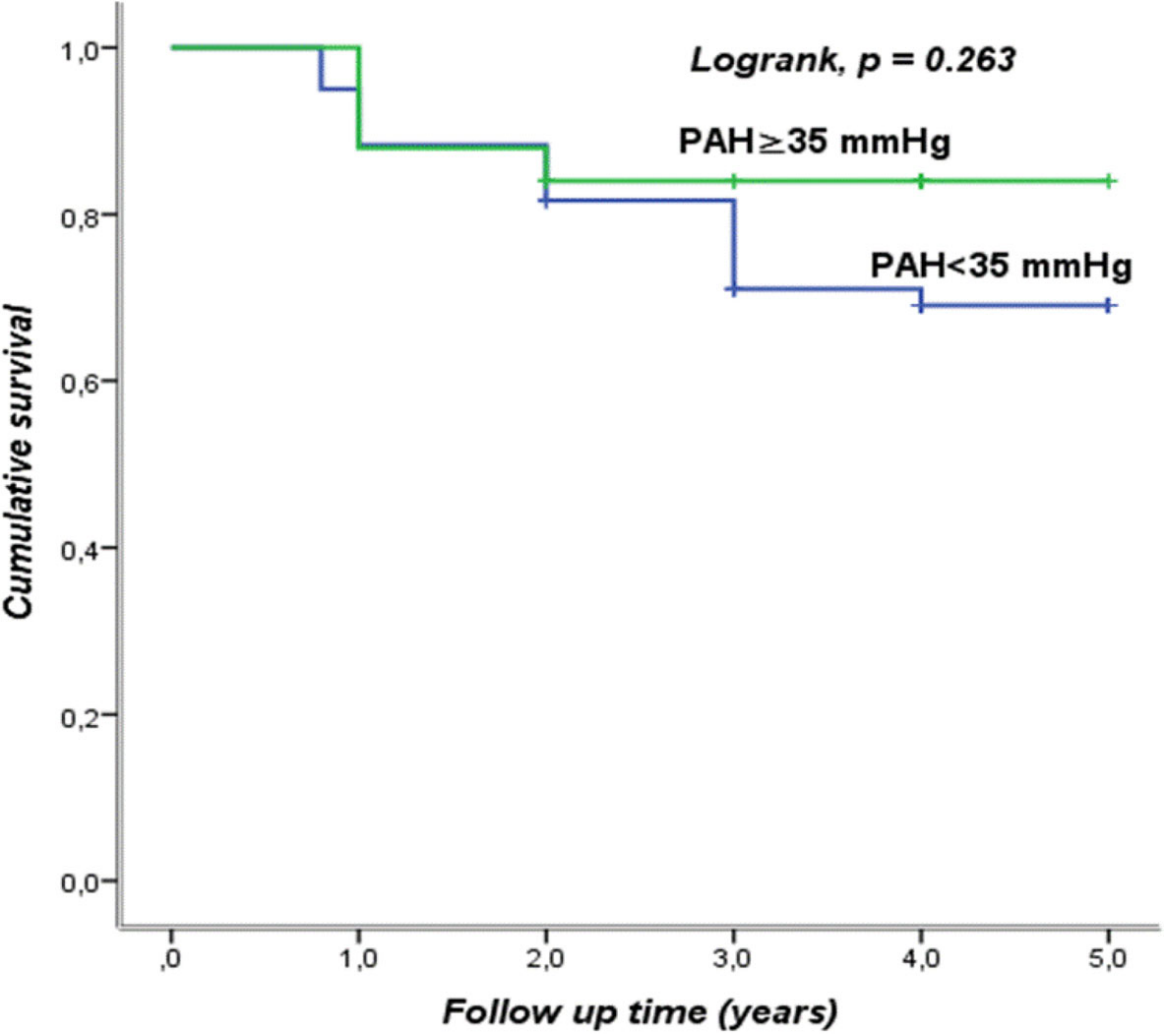
Survival curve

During the 3 years of follow-up, 4 out of 25 patients with PAH (16%) had died compared to 18 out of 60 (30%) without PAH and there was no statistically significant difference between the two groups with a *p* = 0.263 (Fig. 2).

## Discussion

The main findings of the present study are as follows. First, roughly 3 out of 10 patients on maintenance hemodialysis had PAH. Second, arrhythmia, vascular access change, diastolic dysfunction and no secure healthcare funding emerged as the main factors independently associated with PAH.

The prevalence of PAH in this study is 29.4%. This value is within the range of 27–57% reported by most studies [20, 21] and similar to that described by Amin et al. [13] in Egypt although echocardiography was performed within 4 h after the end of dialysis. However, it is lower than the value of 58.6 and 60% found by Fabian in Italy [22] and Hayati et al. [12] in Iran, respectively, probably, because the latter survey used an average PAP of 25 mmHg to define PAH. The discrepancy between studies in PAH frequency may probably be due to the differences of PAH definition. The criteria of selection and the time to perform the echocardiography relative to hemodialysis session. In this regard, the high PAH frequency reported by Hayati et al. [23] could be explained by the use of an average PAP of 25 mmHg to define PAH. Having retained the same definition criteria as Ramasubbu et al. [12], namely a systolic PAP ≥ 35 mmHg, the differences in the sample used and the methodology applied were the reasons for having a low prevalence compared to 47% of their series. The time of echocardiography realization and the importance of interdialytic weight gain (IWG) could also influence the prevalence of PAH. Similar to our study, echocardiography was performed within 24 h after the end of dialysis [15]. When pulmonary hypertension was defined as systolic pulmonary arterial pressure (sPAP) ≥ 45 mmHg, the frequency of PAH was only 20% [12]. This may justify our high prevalence compared to the value of 16% reported by Agarwal et al. [6].

Possible explanations of PAH in long term hemodialysis patients can be grouped into 3 categories: 1) a volumetric overload caused by increased cardiac output following an arteriovenous fistula, anemia or hypervolemia [24, 25]; 2) barometric overload, a consequence of the increase in pulmonary vascular resistance caused by endothelial dysfunction induced by uremia, pulmonary embolism, calcification of the pulmonary artery or other comorbid diseases,

including pulmonary disease chronic obstructive or connective tissue disease, and 3) an increase in pulmonary capillary pressure caused by systolic and diastole in heart failure [8]. Some authors have claimed that microbubbles originating from dialyzers can also become embedded in the pulmonary blood circuit and cause an increase in pressure in the pulmonary circulation [26].

The independent determinants of pulmonary arterial hypertension in our study were unsecured funding for dialysis, arrhythmia, vascular access first change, and diastolic dysfunction. The positive association of unsecured funding (due to low socioeconomic status) with PAH reflects irregular dialysis sessions with subsequent significant interdialytic weight gain through renal sodium and water retention [22]. Sodium and water retention and subsequent hemodilution may also explain hyponatremia that may explain its significant difference in patients with PAH, although it did not persist after adjusting for funding, hyponatremia and arrhythmia. The increase in free water due to the increase in antidiuretic hormone (Vasopressin) can also be responsible for hyponatremia [19]. The vascular access change, most often from catheter to more efficient vascular access such as arteriovenous fistula aggravates the pulmonary congestion through increased circulating blood volume. This lack of care is also the cause of a late referral, justifying the fact that several patients start hemodialysis in disaster with a temporary catheter that they find it difficult to change after lack of financial sources. Indeed, Indeed, Arterio-venous fistula, the ideal approach in hemodialysis, associated with anemia and hypervolemia largely contribute to increasing cardiac venous return, which in turn will lead to a barometric increase in the pulmonary artery [27, 28]. There are many parameters that participate in diastolic heart failure, among which long-term arterial hypertension in chronic dialysis patients is the basis of an increase in left ventricular mass, a defect in relaxation of the left ventricle with elevated blood pressure filling. The loss of vascular elasticity and the proliferation of myocytes found in many diabetic patients is also one of the major causes [29]. Diastolic dysfunction, with a rise in filling pressures resulting in post-capillary PAH, could also explain the observed association with PAH. Our finding showing an association between diastolic dysfunction and PAH corroborate with that reported by Abdelwhab et al. [11], Abedini et al. [29] and Agarwal [6]; the latter found an increased left atrium diameter. The finding of our study point out the role of volumetric overload in the pathogenesis of PAH among maintenance hemodialysis patients. Other studies identified increased age [12], female [13], lower body mass index [12], high cardiac output [25], lower hemoglobin [24], lower metabolites of nitric oxide [25], upper dialysis cycle [22], and lower diastolic blood pressure [22] as additional factors associated with PAH.

In the present study patients with PAH had elevated E/E ratio and ProBNP level suggesting a chronic volume overload and subsequent diastolic dysfunction [9]. This chronic volume overload translated in increased inferior vena cava diameter at exhalation that could be the probable cause of an association between an elevated right atrial diameter and PAH. Chronic volume overload was more frequent in patients with suboptimal dialysis and subsequent increased interdialytic weight gain related to irregular dialysis sessions favored by unsecured funding. In fact, in previous studies, a decrease in PAP following short AV fistula compression [24], as well as fluid removal, has been reported [30]. The use of the diuretics and inhibitors of Renin Angiotensin Aldosterone system may therefore be associated with lower pulmonary hypertension (although we did not find a difference in LV mass index between groups). Except from abovementioned hemodynamic factors, biochemical factors could also contribute to PAH in maintenance hemodialysis patients. In this regard, an increase in circulating nitric oxide synthase inhibitors like asymmetric dimethyl arginine (ADMA) accumulate with subsequent poor availability of nitric oxide, oxidative stress, endothelial dysfunction and vasoconstriction that can worsen PAH [25]. Increased stiffness of the pulmonary capillaries due to hyperparathyroidism and pulmonary vascular calcification is one possible explanation for the occurrence of PAH [31]. As expected, we did not establish an association between pulmonary arterial hypertension, calcium, phosphorus and parathyroid [20, 32]. That is because our patients were younger, with lesser duration of HD, cardiovascular diseases and comorbidities than those published elsewhere.

### Study limitations

The interpretation of the results of the present study should take into account of some limitations. First, the cross-sectional design of the study precludes the establishment of temporal relationship between the issues of interest. Second, the small sample size did not allow sufficient power to statistical tests to identify potential associations between variables of interest. Third, the unique measurement of parameters of interest could have over- or underestimated their true values. Fourth, computerized tomography was not performed to exclude other potential causes of PAH.

## Conclusion

PAH, was a common clinical finding in the present case series and affects more than one in four patients on maintenance hemodialysis in sub-Saharan African.

## Data Availability

The datasets used and/or analysed during the current study are available from the corresponding author on reasonable request.

## Abbreviations

CHF: Congestive Heart Failure
DM: Diabetes Mellitus
EF: Ejection Fraction
ESRD: End Stage Renal Disease
GFR: Glomerular Filtration Rate
HD: Hemodialysis
HT: hypertension
IVC: Inferior Vena Cava
IVS: Interventricular Septum
LV: Left Ventricle
LVEDD: Left Ventricle End Diastolic Diameter
LVESD: Left Ventricle End Systolic Diameter
LVH: Left Ventricular Hypertrophy
LVMi: Left Ventricular Mass index
LVPW: Left Ventricular Posterior Wall
RUV: Residual urination volume
PAH: Pulmonary Arterial hypertension
sPAP: systolic Pulmonary Arterial Pression
